# Primary hyperparathyroidism in Saudi Arabia revisited: a multi-centre observational study

**DOI:** 10.1186/s12902-022-01059-7

**Published:** 2022-06-09

**Authors:** Yousef Al-Saleh, Abdullah AlSohaim, Reem AlAmoudi, Ali AlQarni, Raed Alenezi, Layla Mahdi, Hend Alzanbaqi, Samah M. Nawar, Hibah AlHarbi, Abdulrhman ALMulla, Maryam Al Qahtani, Salih Bin Salih, Faisal Al Anazi, Najla Saleh, Seham Saleh, Ali AlAklabi, Shaun Sabico, Nasser M. Al-Daghri

**Affiliations:** 1 College of Medicine, King Saud Bin Abdulaziz University for Health Sciences, 22490 Riyadh, Saudi Arabia; 2 King Abdullah International Medical Research Center, Riyadh, 11481 Saudi Arabia; 3 Department of Medicine, King Abdulaziz Medical City, Riyadh, Ministry of National Guard-Health Affairs, Riyadh, 14611 Saudi Arabia; 4 Chair for Biomarkers of Chronic Diseases, Biochemistry Department, College of Science, King Saud University, Riyadh, 11451 Saudi Arabia; 5 College of Medicine, King Saud Bin Abdulaziz University for Health Sciences, Jeddah, Saudi Arabia; 6 King Abdullah International Medical Research Center, Jeddah, Saudi Arabia; 7 Department of Medicine, King Abdulaziz Medical City, Jeddah, Ministry of National Guard-Health Affairs, Jeddah, Saudi Arabia; 8 King Saud Bin Abdulaziz University for Health Sciences, Al Ahsa, Saudi Arabia; 9 King Abdullah International Medical Research Center, Al Ahsa, Saudi Arabia; 10 Department of Medicine, King Abdulaziz Hospital, Ministry of National Guard Health Affairs, Al Ahsa, Saudi Arabia; 11 Present Address: Department of Medicine, Johns Hopkins Aramco Health Care, Dhahran, Saudi Arabia; 12 Department of Medicine, College of Medicine, Majmaah University, AlMajmaah, 11952 Saudi Arabia; 13 Prince Sultan Cardiac Center, Riyadh, Saudi Arabia

**Keywords:** Primary hyperparathyroidism, Parathyroid hormone, Calcium, Saudi Arabia

## Abstract

**Purpose:**

Primary hyperparathyroidism (PHPT) is a common cause of hypercalcemia and remains understudied within the Arabian population. The present study, the largest of its kind within the Gulf Cooperation Council (GCC) countries, aims to determine the demographics and clinical presentation of PHPT in Saudi Arabia.

**Methods:**

In this multi-center retrospective study involving three tertiary hospitals in different geographic locations of Saudi Arabia namely, Riyadh, Al Ahsa and Jeddah, a total of 205 out of 243 confirmed PHPT cases aged 16 to 93 years old were included (*N* = 96 from Riyadh; *N* = 59 from Al Ahsa and *N* = 50 from Jeddah). Demographics, clinical manifestations and surgical outcomes were recorded as well as laboratory and radiologic investigations including serum parathyroid hormone (PTH), 25(OH)D, adjusted calcium, estimated glomerular filtration rate (eGFR) and nuclear scan outcome.

**Results:**

PHPT cases appeared to increase over time when compared to other local studies published so far, with 12.8 cases per 100,000 hospital population. Females outnumber males (3:1) with 86% seen as out-patients. The average age was 59.8 ± 15.5 years. Abnormal PTH scan was seen in 171 patients (83.4%). Kidney stones was the most common renal manifestation (32 cases, 15.6%) and osteoporosis was the most common skeletal manifestation (67 cases, 32.7%). Al Ahsa had the highest prevalence of multiple comorbidities at 54% and the highest prevalence of obesity as a single comorbidity (17%) compared to other regions (*p* < 0.05). Jeddah recorded the highest prevalence of osteoporosis with bone and joint pains (30%) (*p* < 0.05).

**Conclusion:**

Comparison of present data with previous local studies suggest an increasing trend in PHPT cases in Saudi Arabia. Regional variations in the clinical presentation of PHPT were observed and warrant further investigation.

## Mini abstract

Primary hyperparathyroidism (PHPT) is an understudied topic in Saudi Arabia. The present study reported confirmed cases of PHPT across major cities in Saudi Arabia and found not only an increased trend compared to previous studies spanning 2 decades of local data but also regional variations in terms of clinical presentations.

## Introduction

Primary hyperparathyroidism (PHPT) is a common endocrine disorder and is believed to be the most common cause of hypercalcemia. It mainly affects the middle-aged, elderly populations and women by as much as two to three times more frequent than men [1]. In a multi-racial study of 15, 234 patients, Yeh and colleagues found the incidence of PHPT to be highest among blacks followed by whites, with incidences from Asians and Hispanics considerably lower than whites [2]. Aside from age, sex and ethnicity, genetic factors such as polymorphisms of the calcium sensing receptor (CASR) gene have been observed to increase susceptibility to PHPT, especially in Caucasians, based on a meta-analysis of 202 case–control studies involving 693 PHPT patients and 1252 controls [3]. A major complication of PHPT and hyperparathyroidism (HPT) in general are skeletal disorders such as osteoporosis and increased risk for fragility fractures [4]. Currently, treatment for PHPT is mostly accomplished surgically with good outcome and prognosis [5].

In the Middle-East, specifically in Saudi Arabia (SA), there is a dearth of evidence of studies of PHPT. In one single-center retrospective study from the central region published in 1999, only 24 cases of PHPT (21 females and 3 males) covering a 16-year period (1982–1997) were documented [6]. The prevalence of PHPT was at 11.3 cases per 100,000 hospital population, with hypercalcemia being the most common feature (92%) and caused by single parathyroid adenoma in the majority of patients (85%) [6]. Another retrospective study from the same center was updated in 2007 and had 46 cases documented from 2000–2006 (35 females and 11 males) [7]. Bone pains were observed in 46% of the cases and was the most common symptom at presentation followed by asymptomatic cases (24%), renal stones (15%), polyuria (6%), and depression and/or constipation (4%) [7]. Within the gulf region, only one similar study was published in Qatar involving 161 patients (111 females and 50 males) gathered from 1995–2014 [8]. PHPT was the most common form of HPT (67.7%) and abnormal PTH glands were detected in 70% of cases following Sestamibi-^99m^Tc (MIBI) scintigraphy [8]. Aside from the studies mentioned, no other follow-up studies from SA were documented to monitor trends.

In order to fill this gap and to provide an updated evidence on PHPT which is lacking in the region, the present multi-center study aimed to examine the demographics, mode of presentation, work-up and complications of PHPT in SA, with 3 participating major tertiary hospitals covering the 3 major regions in the country.

## Methods

### Study design and participants

In this observational multi-center study, a retrospective analysis of confirmed PHPT cases from 2016–2018 was carried out in three major tertiary hospitals supervised by the Ministry of National Guard Health Affairs (MNGHA), SA. These hospitals are located in Riyadh, Al Ahsa and Jeddah, representing the central, eastern and western regions of the country, respectively. All these centers share a common database of electronic medical records, laboratory and imaging systems managed by MNGHA. All the methods in the present study were carried out in accordance with the 1975 Declaration of Helsinki.

### Data collection

Eligible cases were searched in the electronic medical records using the following keywords: primary hyperparathyroidism, hyperparathyroidism, hypercalcemia, parathyroid adenoma, parathyroid cancer, parathyroidectomy and parathyroid surgery. Data retrieved included demographics (age and sex), medical history, mode of presentation, clinical manifestations, comorbidities and investigations. Laboratory investigations recorded, if available, included 24-h urine test for Ca, serum intact PTH, adjusted calcium, 25(OH)D and estimated glomerular filtration rate (eGFR), which was calculated using the Cockroft and Gault formula [9]. For the purpose of this study, the average of 3 serum PTH and adjusted calcium measurements prior to surgery were used. The last 2 serum 25(OH)D levels were averaged and the mean was included in this analysis. Succeeding biochemical assessments were done at 6 weeks, 3 months and 6 months post-surgery. Unsuccessful surgeries were defined as persistence of hypercalcemia and PTH (elevated adjusted calcium and PTH) 6 months after operation. Assessment of serum intact PTH in MNGHA was measured using 3^rd^ generation assays. All MNGHA laboratories are certified by the College of American Pathologists (CAP). Results of confirmatory imaging investigation in the form of Sestamibi-^99m^Tc (MIBI) scintigraphy were collected. PHPT is confirmed if the patient has elevated adjusted serum calcium (Ca) levels with increased PTH levels, following the 2014 classification and guidelines by Bilezikian and colleagues [10, 11].

### Data analysis

Data was analyzed using SPSS version 22 (IBM, Chicago, IL, USA). Categorical variables were presented as frequencies and percentages (%). Continuous variables were presented as mean ± standard deviation (SD) for those with normal distribution. Non-normal variables such as all laboratory variables were presented as mean ± standard error (SE). None normally distributed data were presented as mode and interquartile range Chi-square test was performed to compare categorical variables while Kruskal–Wallis H test was done to compare continuous variables. A p-value < 0.05 was considered statistically significant.

## Results

### Characteristics of patients

Primary search of the electronic medical records of MNGHA from 2016–2018 (*N* = 1,603,000 active and inactive files) using the search key words revealed 243 cases (*N* = 120 Riyadh, *N* = 68 Al Ahsa and *N* = 55 Jeddah) or 12.8 cases per 100,000 hospital population. Out of the 243, 38 cases (15.6%) were excluded for the following reasons; 7 were duplicate cases, 29 had no imaging tests done, 1 patient had active cancer and 1 was considered corrupted file. All in all, 96 cases included were from Riyadh, 59 from Al Ahsa and 50 from Jeddah. Those with HPT from secondary causes (e.g., vitamin D deficiency and chronic kidney failure) and non-Saudis, were excluded.

Table 1 shows the demographic and clinical features of all patients according to region. Gender distribution showed 163 (79.5%) and 42 (20.5%) were females and males, respectively. Majority (86%) were seen as out-patients and only 7 cases (3.7%) in emergency settings. The remaining 10.3% were seen as in-patients. The mean age was 59.8 ± 15.5 years. The youngest patient was 16 years old and the oldest was 93 years. Abnormal PTH scan was seen in 171 patients (83.4%). Medical history revealed that more than half of the patients have multiple comorbidities (50.2%), with obesity being the most common single comorbidity (9.8%), followed by hypertension (5.4%) and diabetes mellitus (4.9%). Hypertension was the most common comorbidity in Riyadh (7%) while obesity was the most common comorbidity in both Al Ahsa (17%) and Jeddah (12%). Only 6 participants recorded the presence of thyroid nodules in all participants, 5 of whom were in Riyadh. Thirty-two patients (15.6%) had no comorbidities and when stratified according to region, Jeddah had the most participants with no comorbidities (28%) followed by Riyadh (14%) and Al Ahsa (8%). Among the clinical features on admission, the presence of kidney stones was the most common renal complication as documented in 32 cases (15.6%) followed by hypercalciuria and polyuria, both at 4.4%. Osteoporosis was the most common skeletal manifestation observed in 67 cases (32.7%) followed by bone and joint pains (16.6%). Thirty-one cases (15.1%) have both cortical bone loss as well as bone and joint pains. Twenty-two (10.7%) patients presented with multiple gastrointestinal manifestations, with abdominal pain (7.3%) and constipation being the most common. Among neurologic/psychological features, 17% of cases showed weakness and fatigue with only 5 cases (2.4%) having depression. A total of 18 cases (8.8%) presented with both neuromuscular and psychologic manifestations. Hypertension on admission was the most common cardiovascular manifestation seen in 90 patients (43.9%) and 18 (8.8%) had multiple cardiovascular symptoms (Table 1).

**Table 1:** Clinical characteristics of patients

Parameters	All	Riyadh	Al Ahsa	Jeddah
N	205	96	59	50
Age (years)	59.8 ± 15.5	62.6 ± 15.2	59.0 ± 15.7	55.3 ± 14.9^*!^
Female	163 (79.5)	84 (87.5)	42 (71)	37 (74)
**Mode**				
ER	7 (3.7)	3 (4)	0	4 (8)
In-patient	19 (10.1)	4 (5)	14 (25)	1 (2)
Out-patient	162 (86.2)	77 (92)	43 (75)	42 (89)
**Positive for PTH scan**	171 (83.4)	77 (80)	51 (86)	43 (86)
**Medical History**				
Obesity	20 (9.8)	4 (4)	10 (17)	6 (12)
Diabetes Mellitus	10 (4.9)	4 (4)	4 (7)	2 (4)
Dyslipidemia	2 (1.0)	1 (1)	0	1 (0)
Hypertension	11 (5.4)	7 (7)	4 (7)	0
Hypothyroidism	5 (2.4)	4 (4)	1 (2)	0
Thyroid nodule	6 (2.9)	5 (5)	0	1 (2)
Multiple	103 (50.2)	49 (51)	32 (54)	22 (44) ^*!^
Others	16 (7.8)	9 (9)	3 (5)	4 (8)
None	32 (15.6)	13 (14)	5 (8)	14 (28)
**Clinical manifestations on admission**				
**Renal**				
Hypercalciuria	9 (4.4)	3 (3)	2 (3)	4 (8)
Kidney Stones	32 (15.6)	15 (16)	11 (19)	6 (12)
Polyuria	9 (4.4)	5 (5)	1 (2)	3 (6)
None	154 (75.6)	73 (76)	45 (76)	36 (72)
**Skeletal**				
Osteoporosis	67 (32.7)	42 (44)	16 (27)	9 (18) ^*^
Bone and joint pain	34 (16.6)	8 (8)	14 (24)	12 (24)
Both	31 (15.1)	8 (8)	8 (14)	15 (30)
None	73 (35.6)	38 (40)	21 (36)	14 (28)
**Gastrointestinal**				
Abdominal pain	15 (7.3)	5 (5)	8 (14)	2 (4)
Anorexia	5 (2.4)	1 (1)	3 (5)	1 (2)
Constipation	10 (4.9)	3 (3)	1 (2)	6 (12)
Nausea/Vomiting	2 (1.0)	1 (1)	1 (2)	0
Peptic Ulcer	1 (0.5)	0	0	1 (2)
Multiple symptoms	22 (10.7)	4 (4)	8 (14)	10 (20) ^*^
None	150 (73.2)	82 (85)	38 (64)	30 (60)
**Neuromuscular and Psychologic**				
Weakness/fatigue	35 (17.1)	6 (6)	12 (24)	15 (30)
Depression	5 (2.4)	2 (2)	2 (3)	1 (2)
Multiple symptoms	18 (8.8)	2 (2)	3 (5)	13 (26)^*!^
None	147 (71.7)	86 (90)	40 (68)	21 (42)
**Cardiovascular**				
Hypertension	90 (43.9)	49 (51)	21 (36) ^*^	20 (40) ^*^
LVH	1 (0.5)	1 (1)	0	0
Bradycardia	2 (1)	1 (1)	1 (2)	0
Multiple symptoms	18 (8.8)	5 (5)	13 (22)	0
None	94 (45.9)	40 (42)	24 (41)	30 (60)
**Single**				
Left lower lobe	6 (2.9)	6 (6)	0 ^*^	0 ^*^
Left upper lobe	68 (33.2)	27 (28)	24 (41)	17 (34)
Right lower lobe	72 (35.1)	31 (32)	25 (42)	16 (32)
Right upper lobe	10 (4.9)	7 (7)	0	3 (6)
None	49 (23.9)	25 (26)	10 (17)	14 (28)
**Bilateral**	15 (7.3)	6 (6)	2 (3)	7 (14)

Note: Data presented as mean ± SD for continuous and N (%) for frequencies; “*” denotes significance compared to Riyadh and “!” denotes significance compared to Dammam; significant at *p* < 0.05

With regards to the possible presence of adenomas, scintigraphy findings showed that 156 patients overall (76.1%) had solitary findings. The right lower lobe of the thyroid was the most common site with 72 cases (35.1%) followed by the left upper lobe with 68 cases (33.2%). Both the right lower lobe and the left upper lobe were also the most common sites for solitary masses suggestive of adenoma seen in all regions. Bilateral was seen in 15 cases (7.3%) (Table 1). In all patients, only one had findings suggestive of hyperplasia and this was recorded in Jeddah.

When stratified according to region, patients seen in Jeddah had the lowest mean age at 55 years and were significantly the youngest as compared to Al Ahsa (59 ± 15.7 years) and Riyadh (62.6 ± 15.2 years) (*p*—values < 0.05). No significant differences were seen in sex distribution as well as mode of consultation and prevalence of abnormal PTH scan. Al Ahsa had the highest prevalence of multiple comorbidities at 54% and the highest prevalence of obesity as a single comorbidity (17%) compared to other regions. The prevalence of renal manifestation was comparable in all regions. In contrast, prevalence of osteoporosis was highest in Riyadh at 44%, while Jeddah recorded the highest prevalence of combined osteoporosis with bone and joint pains (30%) (*p* < 0.05). Jeddah recorded the highest prevalence of multiple gastrointestinal symptoms at 20% as well as constipation (14%) compared to other regions (*p* < 0.05). Jeddah also had the highest prevalence of weakness and fatigue (30%) as well as combined neuromuscular and psychologic symptoms (26%) which was statistically significant compared to Al Ahsa and Riyadh (*p*-values < 0.05). Riyadh on the other hand, had the highest prevalence of hypertension on admission (51%) (*p* < 0.05) and was the only region to have recorded all cases of single left lower lobe adenoma. There was no difference in the prevalence of bilateral adenoma across regions (Table 1).

### Laboratory investigations

Mean laboratory investigations conducted are presented in Table 2. Elevated PTH levels were seen in all patients (mean ± SD = 30.0 ± 2.0 pg/ml) as well as mean adjusted calcium (2.8 ± 0.12 mmol/L). Worthy to note is that circulating PTH and adjusted calcium presented were averages taken from 3 follow-up visits. Mean serum 25(OH)D was borderline insufficient (50.2 ± 1.8 mmol/L) and mean eGFR was 86.3 ± 1.7 mL/min/1.73m^2^ (Table 2). When stratified according to regions, Jeddah recorded the highest average PTH levels and was statistically significant compared to Al Ahsa and Riyadh (*p* < 0.001). Jeddah also had the lowest mean 25(OH)D which was well within the deficient range (42.8 ± 3.2 nmol/L). There were no statistically significant differences elicited across regions in terms of mean adjusted Ca and eGFR (Table 2).

**Table 2:** Laboratory investigations done

Parameters	All	Riyadh	Al Ahsa	Jeddah
N	205	96	59	50
PTH (pg/ml) *	30.0 ± 2.0	24.1 ± 1.7	33.1 ± 4.4	37.5 ± 5.0^*!^
Adjusted Ca (mmol/L)*	2.8 ± 0.12	2.7 ± 0.02	3.1 ± 0.4	2.7 ± 0.03
25 (OHD) (nmol/L)	50.2 ± 1.8	55.4 ± 2.9	48.1 ± 2.9	42.8 ± 3.2^*^
eGFR (mL/min/1.73m^2^)	86.3 ± 1.7	82.9 ± 2.2	87.1 ± 3.0	92.2 ± 3.8

**Note**: Data presented as mean ± SE; “*” denotes significance compared to Riyadh and “!” denotes significance compared to Dammam; significant at *p* < 0.05

### Management and outcomes

Table 3 shows the management performed in all patients and across regions. Surgery was done in 86 cases (41.2%) based on surgical guidelines and 59 (68.6%) achieved cure with normal postoperative adjusted Ca and PTH levels. Almost a third of the surgical cases (*N* = 26) were performed in patients below 50 years, indications of which were evidence-based [11]. Failed surgery was recorded in 10 cases (4.9%) and second surgery was performed in only 2 cases (1%). Across regions, Al Ahsa had the highest number of surgeries performed with 34 (58%) cases (*p* < 0.05) while Riyadh had the highest number of failed surgeries with 7 (7%) cases (*p* < 0.05). Only Jeddah registered no case for second surgery (*p* < 0.05) (Table 3).

**Table 3:** Management and outcomes of patients

Parameters	All	Riyadh	Al Ahsa	Jeddah
N	205	96	59	50
Surgery Done (yes)	86 (41)	29 (30)	36 (61) ^*^	21 (42) ^*^
Surgery done among those < 50y	26 (30)	9 (30)	8 (22)	9 (43) *
*Successful with normal Ca and PTH*				
No	27 (31)	9 (31)	16 (44) ^*^	2 (10)^!^
Yes	59 (69)	20 (69)	20 (56)	19 (90)
*Second Surgery Performed (N)*	2	1	1	0

**Note**: Data presented as N (%) for frequencies; “*” denotes significance compared to Riyadh and “!” denotes significance compared to Dammam; significant at *p* < 0.05

### The surge in PHPT in KSA

**Fig. 1:**
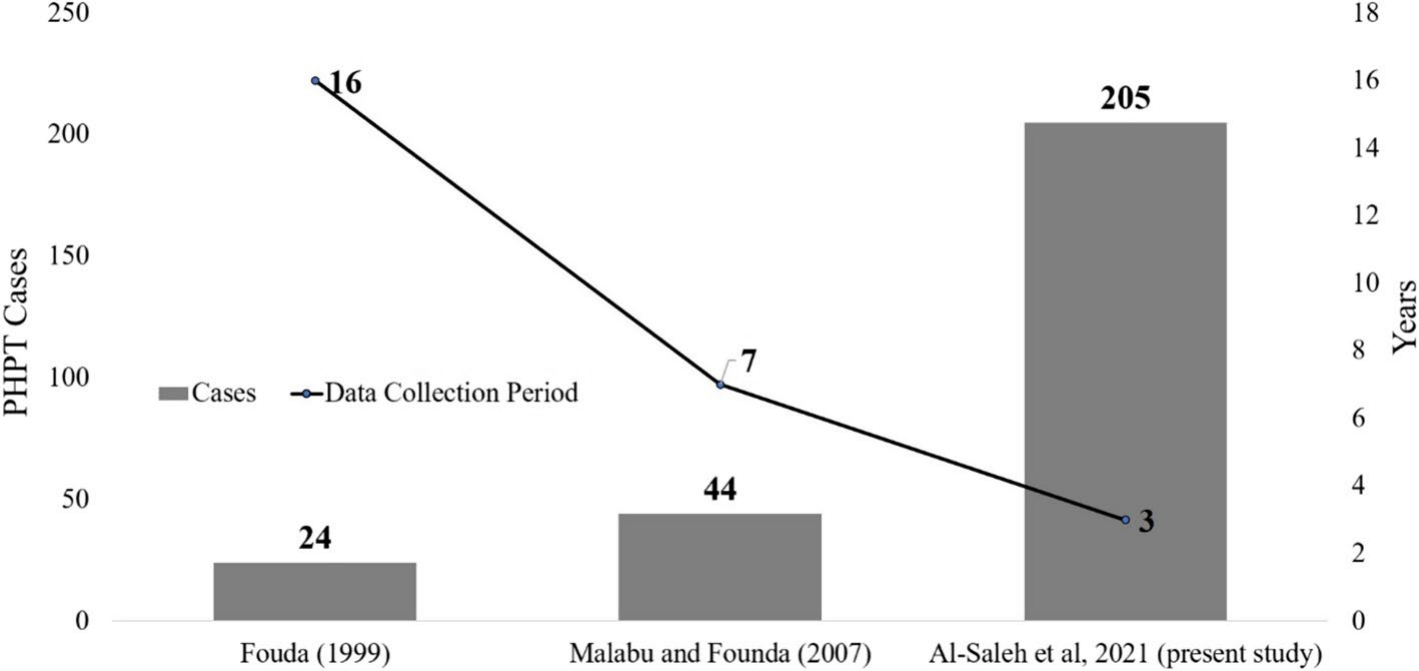
Incidence of PHPT in Saudi Arabia overtime

Lastly, Fig. 1 shows the relative increase in the number of PHPT cases overtime based on the previous and limited studies in Saudi Arabia, with only 24 PHPT cases gathered over a 16-year period (1982–1997) [6], to 44 cases gathered over a 7-year period (2000–2006) [7], to the present 205 cases gathered over a 3-year period (2016–2018), noting that the number of cases per region still exceeded the last observation in 2007 (*N* = 96 Riyadh; *N* = 59 Al Ahsa and *N* = 50 Jeddah) (not shown in figure).

## Discussion

This multi-center observational study clearly demonstrated that the incidence of PHPT appears to be in an upward trajectory based on the comparisons from past and limited studies in Saudi Arabia, which was based from 11.3 cases per 100,000 in 1999 over a 16-year time period (1982–1997), to 12.8 cases per 100,000 over a 3-year period (2016–2018). In terms of scale, the present study is also the largest to date in the country and the entire GCC geographical area, covering the 3 major regions of SA. Given the racial homogeneity in this region and the scope covered, the findings are generalizable and can be used as a reference for the Arab ethnicity. The increased incidence in PHPT over time can be attributed to the rapid development in diagnostic procedures, most notably the automation of serum calcium determination, making it a routine biochemical screening that may have identified number of patients having no symptoms (catch up effect) [12]. In the case of the present study, a large proportion of patients presented no clinical manifestations in both renal and skeletal systems with 16.6% having negative PTH scan. The present findings echo a similar progressive rise in PHPT cases in India, where asymptomatic cases rose sharply from 3 to 13% within 25 years (1995–2019) [13]. For comparison within the Gulf Cooperation Council (GCC) countries, only Bahrain has a more recent epidemiological evidence of PHPT cases which is alarmingly higher than the present study at 274 cases per 100,000 (versus 12.8 cases per 100,000) [14].

Despite the homogeneity of ethnicity, it was apparent that regional variations in the clinical presentation of PHPT existed. Findings from Riyadh confirmed previous observations in 2007 from the same region, highlighting osteoporosis as the most common skeletal manifestation [7] as well as the earlier study in 1999 having the same high prevalence of single PTH adenoma [6], with Riyadh being the only region having all the cases of lower left lobe adenomas in the present study. The most common location of single PTH adenoma however across regions is the right lower lobe, which is similar to the findings of the 7-year study of 147 patients in Japan and the more recent investigation of 156 PHPT European patients in Germany [15, 16].

Worthy to note are the clinical presentations in Al Ahsa and Jeddah which have never been previously documented. The region of Jeddah in particular, had significantly younger patients with the lowest prevalence of skeletal symptoms, the highest prevalence of combined neurologic and psychologic symptoms and gastrointestinal manifestations, as well as having the worst levels of PTH and 25(OH)D compared to other regions. Al Ahsa on the other hand had the highest prevalence of unsuccessful normalization of serum Ca and PTH levels post-surgery and the highest mean Ca. While further investigations are warranted to determine these regional discrepancies, it highlights possible environmental and diverse genetic factors at play, considering the heterogeneity of the Arab genetic pool and the high consanguinity rates which varies by region [17, 18]. Furthermore, and considering that patients in Jeddah are much younger with lower 25(OH)D levels compared to its regional counterparts, PHPT may be an important risk factor osteoporosis in that population, as it has been previously suggested that HPT may be a risk factor for osteoporosis and incident fractures in young females and those in the early postmenopausal period, while being protective on trabecular bone among elderly postmenopausal women [19]. The relatively younger population of PHPT cases in Jeddah compared to other regions may explain the higher prevalence of symptomatic cases, since asymptomatic cases are more commonly observed in older patients [14]. Further investigations are needed to clarify this, since other variables such as disease duration and severity of hypercalcemia were not assessed.

In the present study, the ratio of PHPT in women to men was 3:1. This confirms previous findings indicating sexual dimorphism, with being female on its own as a significant risk factor for PHPT [20–23]. This disparity can be explained by the differences in biochemical activity of PTH which becomes even more apparent after menopause [24]. The definitive effect of the menopausal state on the clinical expression of PHPT suggests a critical role of estrogen withdrawal, which consequently plays a major role in osteoporosis, a typical presentation of postmenopausal women with PHPT [19, 25].

### Limitations

The authors acknowledge several limitations. The retrospective approach limits the findings of the study based on what can be retrieved electronically and several missing data, may substantially alter the present findings. Furthermore, in-depth probe to other less common disorders which can nevertheless influence surgical management such as screening for multiple endocrine neoplasia, familial hypocalciuric hypercalcemia and other genetic disorders were not done nor seen in records, as well as other treatment for the remaining patients such as dynamic observation, cinacalcet and/or anti-osteoporosis therapy. Other than the surgical procedure conducted, medications of patients were also not included in the data collection and this could have explained the outcomes. Lastly, more details such as incidental findings of thyroid nodules which was reportedly common prior to PHPT diagnosis [26], as well as data on concordance and discordance from Sestamibi scans which can alter operative management [27], could have added more value to the present study. Such information should be retrieved for future investigations.

Despite the caveats, the present study is the largest of its kind in describing the clinical presentations and outcomes of PHPT patients in Saudi Arabia and the wider GCC region up to our best knowledge. It is also the first to describe regional variations in PHPT within the country. The findings have clinical merit and adds a valuable missing piece in the literature, considering the under representation of the Arab ethnicity in PHPT studies. The findings are also generalizable as it covered the major regions of Saudi Arabia. Lastly, the high prevalence of cortical bone loss as well as bone and joint pains suggest that PHPT may be an emerging silent risk factor for osteoporosis in the country and should be taken into consideration in updating national regional guidelines for osteoporosis where PHPT was not considered [28, 29].

## Conclusion

In conclusion, there is, clearly, an increased trend of PHPT in Saudi Arabia based on previous and present data. Regional variations in the clinical presentations of PHPT are evident, with patients in Al Ahsa and Jeddah needing further investigations. We recommend prospective national study to further explore our findings and determine the true incidence of the disease.

## Data Availability

The datasets used and analyzed during the current study are available from the corresponding author on reasonable request. Data used are medical records of private citizens and can only be accessed if authorized.
